# Comparison of two novel diagnostic criteria for bronchopulmonary dysplasia in predicting adverse outcomes of preterm infants: a retrospective cohort study

**DOI:** 10.1186/s12890-023-02590-6

**Published:** 2023-08-23

**Authors:** Xin Wang, Yang-Ke Lu, Yan-Yan Wu, Da-Peng Liu, Jing Guo, Ming-Chao Li, YingYuan Wang, Rui Li, Xiao-Yuan Zhang, Wen-Qing Kang

**Affiliations:** Neonatal Intensive Care Unit, Zhengzhou Key Laboratory of Newborn Disease Research, Children’s Hospital Affiliated to Zhengzhou University, Zhengzhou, China

**Keywords:** Bronchopulmonary dysplasia, Prognosis, Prediction, Preterm infants

## Abstract

**Background:**

This study aimed to compare the predictive value of two diagnostic criteria for bronchopulmonary dysplasia (BPD) in preterm infants with gestational age (GA) < 32 weeks for death or severe respiratory morbidity at corrected age of 18–24 months.

**Methods:**

In this retrospective cohort study, clinical data from July 2019 to September 2021 were classified by 2018 National Institute of Child Health and Human Development (NICHD) and 2019 Jensen definitions of BPD. Based on the follow-up results, the enrolled population was divided into adverse outcome group and normal outcome group. Logistic regression and receiver operating characteristic (ROC) curve analyses were conducted to explore the risk factors of adverse outcomes and evaluate the predictive value of both diagnostic criteria.

**Results:**

Of 451 infants, 141 (31.3%) had adverse outcomes, which increased with increasing severity of BPD. Logistic regression analysis showed only BPD was an independent risk factor for adverse outcomes in preterm infants. ROC analysis revealed that both diagnostic criteria showed similar predictive values (2018 NICHD definition AUC = 0.771 vs. 2019 Jensen definition AUC = 0.770), with specificities of 93.5% and 96.8%, respectively; however, combining them separately with GA or birth weight did not improve their predictive values.

**Conclusions:**

The two novel definitions of BPD demonstrate similar predictive values in predicting death or severe respiratory morbidity at corrected age of 18–24 months, with higher specificity observed in both.

**Supplementary Information:**

The online version contains supplementary material available at 10.1186/s12890-023-02590-6.

## Background

Bronchopulmonary dysplasia (BPD), the most common chronic lung disease in preterm infants, is a clinical syndrome that disrupts alveolar formation and impairs microvascular development [1]. In addition to genetic predisposition, various prenatal and postnatal factors such as exposure to inflammation, intrauterine growth restriction, postnatal bacterial or viral infection, oxygen supplementation, mechanical ventilation, and malnutrition can aggravate lung injury and impede its repair, ultimately leading to BPD [2–4]. BPD increases healthcare costs, prolongs hospital stays, and contributes to the development of long-term respiratory diseases, growth retardation, and neurodevelopmental disorders. It may even affect their quality of life in adolescence and adulthood [5, 6]. Thus, it is imperative to establish a precise definition for early diagnosis, accurate severity grading, long-term prognosis prediction, and identification of populations requiring intervention.

Since its initial proposal in 1967, the definition of BPD has undergone several changes [7–11]. Over the past 20 years, the National Institute of Child Health and Human Development’s (NICHD) 2001 severity-based classification has been widely used in clinical practice [8]. However, its applicability in clinical practice is limited due to its inability to reflect current ventilation modes [12]. In 2018, NICHD revised the definition to include common noninvasive ventilation modes and detailed division of the fraction of inspired oxygen (FiO_2_) in different respiratory support modalities [10]. Jensen et al. proposed an optimal definition in 2019 based on evidence-based approach to predict respiratory and neurodevelopmental outcomes at 18–26 months’ corrected age [11]. However, its efficacy in predicting long-term outcomes needs further evaluation. In this study, we performed a comparison of the two novel BPD definitions and evaluated the predictive value of each definition for predicting late death or long-term adverse respiratory outcomes in preterm infants.

## Methods

### Subjects

This retrospective cohort study included infants with a gestational age (GA) of < 32 weeks admitted within 28 days of birth to the neonatal intensive care unit of Children’s Hospital Affiliated to Zhengzhou University between July 2019 and September 2021. The exclusion criteria were as follows: (1) severe congenital malformations, chromosomal defects or inherited metabolic disease; (2) death before 36 weeks’ postmenstrual age (PMA). This study has been approved by the Ethics committee of Children’s Hospital Affiliated to Zhengzhou University (2022-H-K39). All guardians/parents gave written informed consent.

### Data collection and follow-up

Data on infants, including their gender, gestational age, birth weight, perinatal clinical information, weight and respiratory support at 36 weeks’ PMA, complications during hospitalization, length of hospital stay, and discharge with oxygen were collected for this cohort study. Extrauterine growth restriction (EUGR) was defined if the weight at 36 weeks’ PMA was below the 10th percentile of the weight of the same sex infant, based on the Fenton growth chart [13]. According to the 2018 NICHD definition [10], a diagnosis of BPD requires not only parenchymal lung disease with radiographic evidence, but also respiratory support and FiO_2_ as shown in Table 1 for at least 3 consecutive days at 36 weeks’ PMA to maintain arterial oxygen saturation at 90-95%. The 2019 Jensen definition categorizes BPD severity solely on the basis of level of respiratory support at 36 weeks’ PMA, irrespective of oxygen therapy [11]. Both the definitions classify BPD into 3 severity grades (Table 1).

All infants were followed up at 18–24 months’ corrected age by specially trained physicians to assess the need for oxygen therapy or respiratory monitoring following initial discharge, as well as the number of hospitalizations due to respiratory diseases before follow-up. All follow-up was completed by March 2023.

**Table 1:** Definitions of BPD

2018 NICHD definition	Grade	IMV	NCPAP, NIPPV, or nasal cannula ≥ 3 L/min	Nasal cannula (1 ~ < 3) L/min or hood O_2_	Nasal cannula < 1 L/min
	I	-	21	22–29	22–70
	II	21	22–29	≥ 30	> 70
	III	> 21	≥ 30	-	-
	III(A)	Death from respiratory causes between 14 days after birth and 36 weeks’ PMA.			
2019 Jensen definition	1	Nasal cannula ≤ 2 L/min			
	2	Nasal cannula > 2 L/min, nCPAP, or NIPPV			
	3	IMV			

IMV, invasive mechanical ventilation; NCPAP, nasal continuous positive airway pressure; NIPPV, noninvasive positive pressure ventilation

### Outcomes

In this study, adverse outcomes were defined as either death or severe respiratory morbidity in the first 18–24 months. The severe respiratory morbidity was at least one of the following: (1) hospitalization for respiratory reasons at ≥ 45 weeks’ PMA (mean corrected age at discharge plus 2 standard deviations for very preterm infants in the last 10 years in our center), (2) continued need for oxygen therapy (nasal catheter oxygen or non-invasive ventilation) or respiratory monitoring (pulse oxygen meter or apnea monitor) at the time of discharge, or (3) rehospitalization for respiratory diseases such as acute bronchitis or pneumonia ≥ 2 times before the end of follow-up.

### Statistical analysis

Non-normally distributed measurement data were presented as median (interquartile range) [M (Q1, Q3)], and intergroup comparisons were performed using the Mann-Whitney U test. Count data were expressed as frequency and percentages (%), and comparison between groups was performed using the chi-square test or Fisher’s exact test. Univariate and multivariate logistic regression models were used to analyze the factors affecting the prognosis of preterm infants. Further, the receiver operating characteristic (ROC) curve and area under the curve (AUC) were generated for each regression model to evaluate their predictive value for adverse events. Delong test was used to compare the AUCs. All statistical analyses were performed with SPSS 26.0 and Medcalc 20.111 software. Statistical significance was set at P < 0.05.

## Results

### Characteristics of the study population

**Fig. 1:**
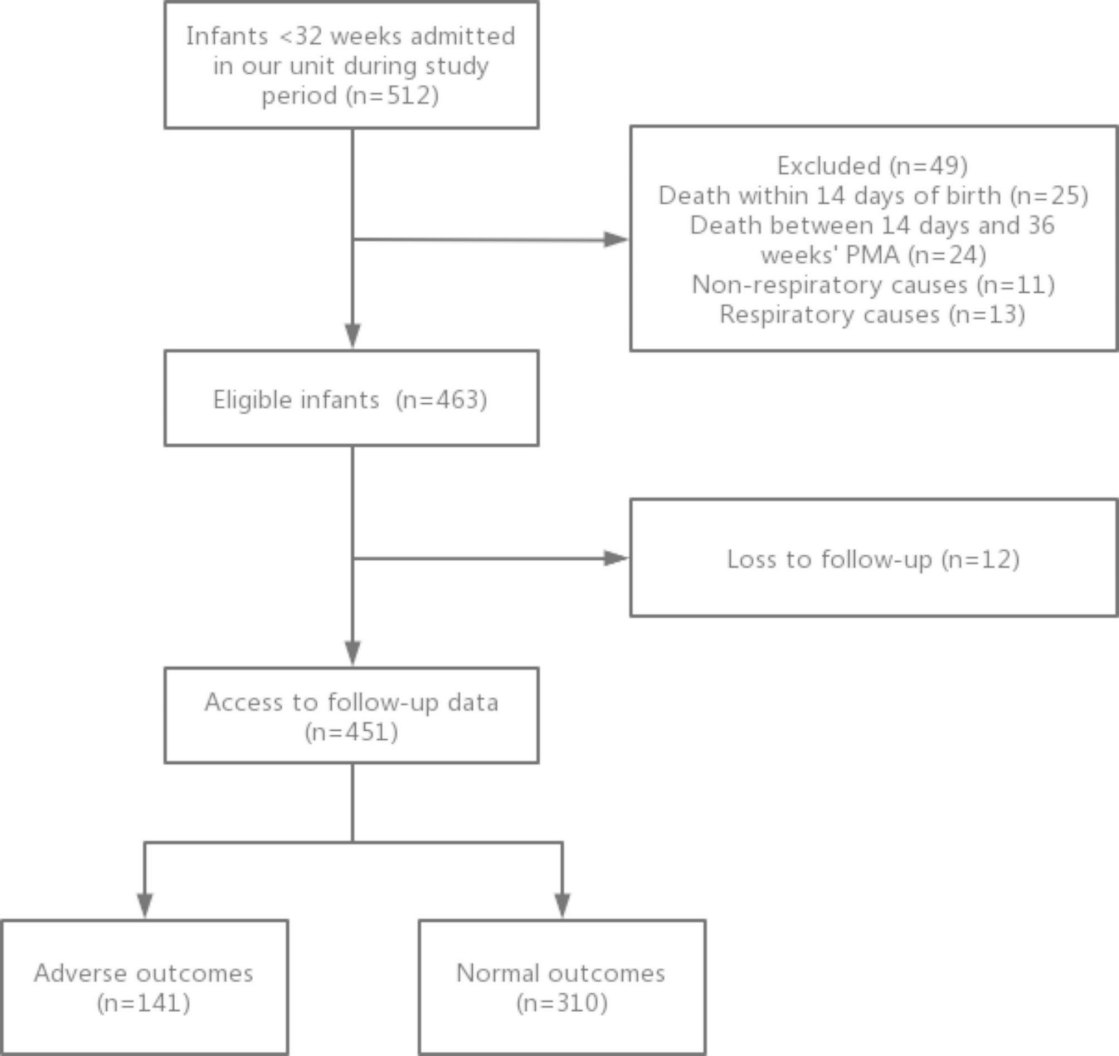
Flow diagram of the study population

A total of 512 preterm infants met the inclusion criteria during the study period (Fig. 1). Among them, 49 died before 36 weeks’ PMA, of whom 11 died from non-respiratory causes and 38 died from respiratory causes. Of the remaining 463 infants, 451 were assessed for the outcomes while 12 were lost to follow-up (According to the 2018 NICHD definition, non-BPD was present in 5 infants, grade I BPD in 1, grade II BPD in 4, and grade III BPD in 2). The gestational age and birth weight were 29.9 (28.4, 31.0) weeks and 1300 (1070, 1540) g, respectively. Based on the follow-up results, 141 (31.3%) infants were assigned to the adverse outcome group, including 120 (85.1%) with BPD; while 310 (68.7%) were in the normal outcome group, of whom 133 (42.9%) had BPD. Fourteen infants died from 36 weeks’ PMA to 18–24 months, with 3 cases of BPD, 9 cases of BPD with pulmonary infection, and 2 cases of BPD with pulmonary hypertension. Of these, 10 infants died during their initial hospitalization, while 4 infants died at rehospitalization due to respiratory diseases after the first discharge.

### Outcome rates according to different diagnostic criteria

Among 451 preterm infants who were followed up, 253 (56.1%) were diagnosed with BPD according to either of the two diagnostic criteria. Using the 2018 NICHD definition, 52 (11.5%) were classified as grade I BPD, 130 (28.8%) as grade II BPD, and 71 (15.7%) as grade III BPD. When applying the 2019 Jensen definition, 159 (35.3%), 54 (12.0%), and 40 (8.9%) cases of grade 1, 2, and 3 BPD were diagnosed, respectively. The frequency of adverse outcomes rose from 10.6% among infants without BPD to 71.8% and 75.0% among those with grade 3 BPD using the 2018 NICHD and 2019 Jensen definitions, respectively. There was a stepwise increase in the incidence of the adverse outcomes as BPD severity increased (Table 2).

**Table 2:** Adverse outcome rates according to different diagnostic criteria

Adverse Outcomes	n = 141	2018 NICHD definition				2019 Jensen definition			
		No BPD (n = 198)	Grade I (n = 52)	Grade II (n = 130)	Grade III (n = 71)	No BPD (n = 198)	Grade 1 (n = 159)	Grade 2 (n = 54)	Grade 3 (n = 40)
Death after 36 weeks’ PMA	14/141 (9.9)	0	1/52 (1.9)	2/130 (1.5)	11/71 (15.5)	0	2/159 (1.3)	2/54 (3.7)	10/40 (25.0)
Hospitalization for respiratory reasons at ≥ 45 weeks’ PMA	26/141 (18.4)	0	2/51^a^ (3.9)	13/128^a^ (10.2)	11/60^a^ (18.3)	0	10/157^a^ (6.4)	8/52^a^ (15.4)	8/30^a^ (26.7)
Oxygen therapy or respiratory monitoring at discharge	38/141 (27.0)	3/198 (1.5)	3/51 (5.9)	17/128 (13.3)	15/60 (25.0)	3/198 (1.5)	16/157 (10.2)	9/52 (17.3)	10/30 (33.3)
Rehospitalization for respiratory diseases≥2 times	99/141 (70.2)	18/198 (9.1)	12/51 (23.5)	38/128 (29.7)	31/60 (51.7)	18/198 (9.1)	41/157 (26.1)	26/52 (50.0)	14/30 (46.7)
Late death or serious respiratory morbidity	141	21/198 (10.6)	15/52 (28.8)	55/130 (42.3)	51/71 (71.8)	21/198 (10.6)	56/159 (35.2)	34/54 (63.0)	30/40 (75.0)

a: The denominators are the total number of nonmortality outcomes

### Comparison of characteristics of preterm infants with different outcomes

Table 3 shows the results of the univariate analysis on the association between general information, perinatal clinical information, weight and the mode of respiratory support at 36 weeks’ PMA, and adverse outcomes. The analysis revealed that GA, birth weight, occurrence of EUGR at 36 weeks’ PMA, treatment with pulmonary surfactant, and BPD were statistically significant (P < 0.05).

**Table 3:** Characteristics of preterm infants with different outcomes

Variables	Adverse outcomes (n = 141)	Normal outcomes (n = 310)	*OR(95%CI)*	*P-value*	Variables	Adverse outcomes (n = 141)	Normal outcomes (n = 310)	*OR(95%CI)*	*P-value*
GA, w, M (Q1, Q3)	29.4(28.0−30.6)	30.0(28.7–31.0)	0.802 (0.709–0.906)	< 0.001	HIP, n(%)	22(15.6)	56(18.1)	0.839 (0.489–1.438)	0.522
30 ≤ GA < 32, n(%)	57(40.4)	164(52.9)	1.00	—	GDM, n(%)	6(4.3)	14(4.5)	0.940 (0.353–2.498)	0.901
28 ≤ GA < 30, n(%)	51(36.2)	107(34.5)	1.371 (0.875–2.150)	0.168	PROM, n(%)	44(31.2)	106(34.2)	0.873 (0.570–1.337)	0.533
GA < 28, n(%)	33(23.4)	39(12.6)	2.435 (1.401–4.232)	0.002	Placental abruption, n(%)	17(12.1)	33(10.6)	1.151 (0.618–2.144)	0.658
BW, g, M (Q1, Q3)	1180(975–1450)	1350(1127–1600)	0.999 (0.998–0.999)	< 0.001	Delivery room resuscitation, n(%)	57(40.4)	111(35.8)	1.217 (0.808–1.831)	0.347
BW ≥ 1500, n(%)	29(20.6)	108(34.8)	1.00	—	2018 BPD, n(%)	120(85.1)	133(42.9)	7.605 (4.543–12.731)	< 0.001
1000 ≤ BW < 1500, n(%)	74(52.4)	164(52.9)	1.680 (1.026–2.752)	0.039	No BPD, n(%)	21(14.9)	177(57.1)	1.00	—
BW < 1000, n(%)	38(27.0)	38(12.3)	3.724 (2.027–6.843)	< 0.001	Grade I BPD, n(%)	15(10.6)	37(11.9)	3.417 (1.612–7.244)	0.001
Male, n(%)	81(57.4)	177(57.1)	1.014 (0.678–1.517)	0.944	Grade II BPD, n(%)	54(38.3)	76(24.5)	5.989 (3.383–10.603)	< 0.001
SGA, n(%)	13(9.2)	23(7.4)	1.267 (0.622–2.581)	0.514	Grade III BPD, n(%)	51(36.2)	20(6.5)	21.493 (10.812–42.724)	< 0.001
EUGR at 36 weeks’ PMA, n(%)	98(74.2)^a^	189(62.2)^b^	1.754 (1.114–2.761)	0.015	2019 BPD, n(%)	120(85.1)	133(42.9)	7.605 (4.543–12.731)	< 0.001
Prenatal corticosteroids, n(%)	107(75.9)	217(70.0)	1.349 (0.855–2.128)	0.198	No BPD, n(%)	21(14.9)	177(57.1)	1.00	—
Treatment with PS, n(%)	95(67.4)	177(57.1)	1.552 (1.022–2.357)	0.039	Grade 1 BPD, n(%)	56(39.7)	103(33.2)	4.583 (2.625−8.000)	< 0.001
Assisted reproduction, n(%)	15(10.6)	18(5.8)	1.931 (0.943–3.953)	0.072	Grade 2 BPD, n(%)	34(24.1)	20(6.5)	14.329 (7.017–29.259)	< 0.001
Cesarean delivery, n(%)	82(58.2)	159(51.3)	1.320 (0.883–1.973)	0.176	Grade 3 BPD, n(%)	30(21.3)	10(3.2)	25.286 (10.844–58.962)	< 0.001

GA, gestational age; BW, birth weight; SGA, small for gestational age; EUGR, extrauterine growth restriction; PMA, postmenstrual age; PS, pulmonary surfactant; HIP, hypertension in pregnancy; GDM, gestational diabetes mellitus; PROM, premature rupture of membranes; BPD, bronchopulmonary dysplasia

a: 132 subjects could measure their body weight at 36 week’ PMA. b: 304 subjects could measure their body weight at 36 week’ PMA.

### Multivariate logistic regression analysis for adverse outcomes in preterm infants

Independent variables with significant differences in the univariate analysis were included as independent variables, along with the 2018 NICHD and 2019 Jensen definitions, while adverse outcomes were used as dependent variables to conduct multivariate logistic regression analysis. The results indicated that only BPD was significantly associated with adverse outcomes (P < 0.05) (Table 4).

**Table 4:** Multivariate logistic regression analysis of risk factors for adverse outcomes combined with different diagnostic criteria

Variables	Joint 2018 NICHD definition		Joint 2019 Jensen definition	
	*OR(95%CI)*	*P-value*	*OR(95%CI)*	*P-value*
GA, w		0.511		0.722
30 ≤ GA < 32	1.00	—	1.00	—
28 ≤ GA < 30	0.703(0.386–1.278)	0.248	0.783(0.432–1.421)	0.421
GA < 28	0.761(0.328–1.764)	0.524	0.859(0.371–1.993)	0.724
BW, g		0.326		0.384
BW ≥ 1500	1.00	—	1.00	—
1000 ≤ BW < 1500	1.439(0.722–2.868)	0.301	1.464(0.732–2.931)	0.281
BW < 1000	2.117(0.793–5.651)	0.134	1.998(0.743–5.373)	0.170
EUGR at 36 weeks’ PMA	1.069(0.598–1.913)	0.821	1.013(0.565–1.817)	0.965
Treatment with PS	1.383(0.831–2.303)	0.212	1.414(0.850–2.351)	0.183
2018 BPD/2019 BPD		< 0.001		< 0.001
No BPD	1.00	—	1.00	—
Grade 1 BPD	3.745(1.712–8.189)	0.001	4.337(2.368–7.945)	< 0.001
Grade 2 BPD	5.375(2.870−10.064)	< 0.001	14.569(6.732–31.527)	< 0.001
Grade 3 BPD	23.961(11.359–50.541)	< 0.001	26.159(10.787–63.436)	< 0.001

GA, gestational age; BW, birth weight; EUGR, extrauterine growth restriction; PMA, postmenstrual age; PS, pulmonary surfactant; BPD, bronchopulmonary dysplasia

### Prediction of the adverse outcomes

**Fig. 2:**
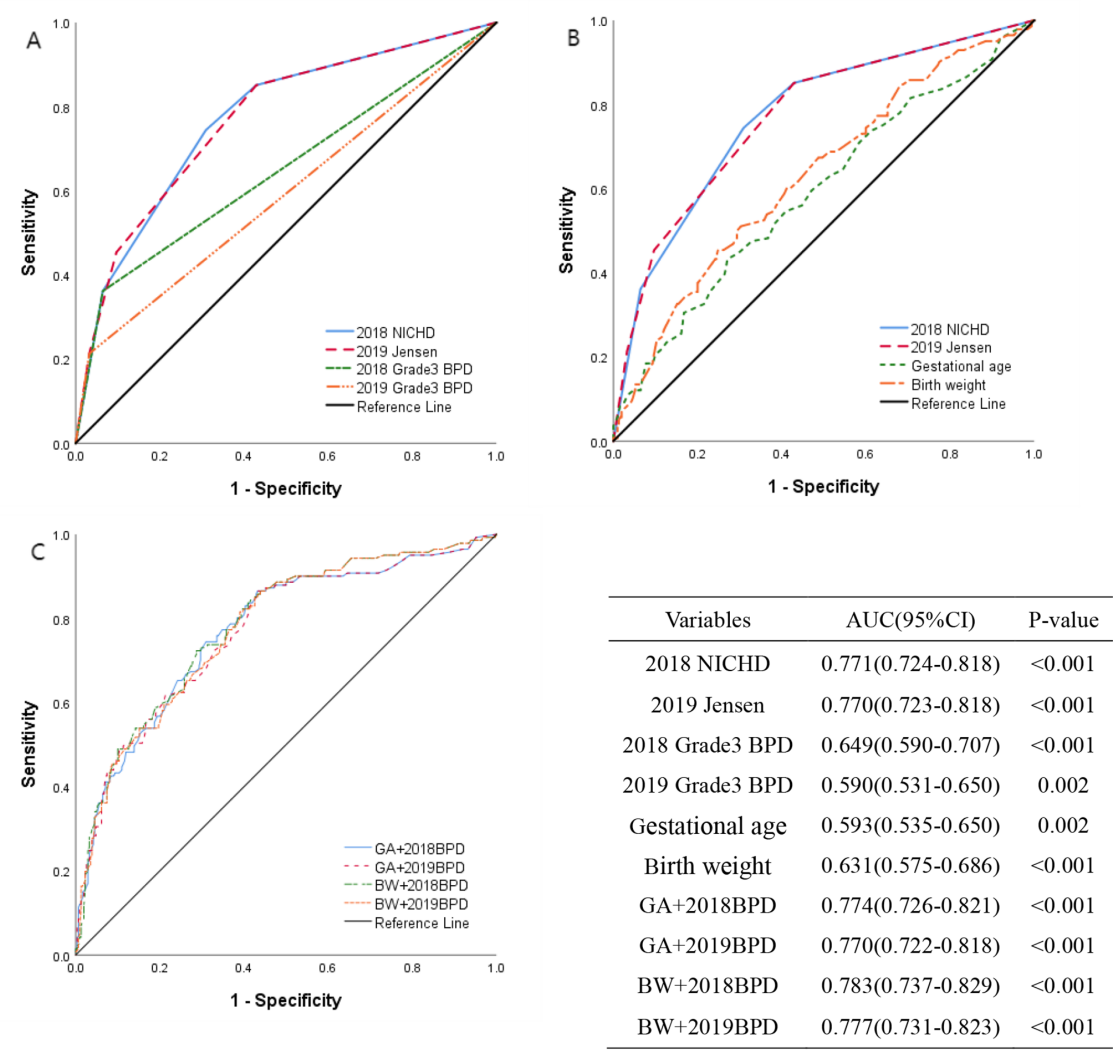
ROC curves for factors associated with the adverse outcomes in A, B and C. A shows the predictive value of the two new diagnostic criteria for adverse outcomes, along with a comparison of the predictive value of grade 3 BPD in the two definitions. B shows the ROC curves of gestational age and birth weight and comparison with the two new diagnostic criteria. C shows the comparison of ROC curves of all the combined model. Table shows areas under the curves and 95% confidence intervals

The ROC curves of the two novel diagnostic criteria and grade 3 BPD in both definitions in predicting adverse outcomes are presented in Fig. 2A. The results revealed that the 2018 NICHD definition (AUC = 0.771) had a sensitivity of 36.2% and a specificity of 93.5% for predicting adverse outcomes, while the 2019 Jensen definition (AUC = 0.770) had a sensitivity of 21.3% and a specificity of 96.8%. There was no statistically significant difference between the AUC values (z = 0.105, P = 0.916). Notably, grade 3 BPD alone was found to have poor efficacy in predicting prognosis (2018 Grade 3 BPD AUC = 0.649 vs. 2019 Grade 3 BPD AUC = 0.590, z = 3.678, P < 0.001). Furthermore, a comparison of the predictive values of the 2001 NICHD definition with that of the 2018 and 2019 definitions was shown in the Additional file 1: Figure S1. The results revealed that the AUC of 2001 NICHD definition was significantly lower than that of 2018 or 2019 definitions (0.730 vs. 0.771, z = 3.958, P < 0.001; 0.730 vs. 0.770, z = 3.241, P = 0.001) (See Additional file 1: Table S1).

Figure 2B and C display the ROC curves for predicting adverse outcomes based on gestational age, birth weight, and their respective combined 2018 or 2019 definitions. The sensitivity and specificity in predicting adverse outcomes by gestational age (AUC = 0.593) were 43.3% and 72.9%, respectively, with an AUC value significantly different from the 2018 NICHD definition (z = 5.525, P < 0.001) and the 2019 Jensen definition (z = 5.423, P < 0.001). Similarly, the sensitivity by birth weight (AUC = 0.631) was 51.1%, with a specificity of 69.7%, and the AUC value differed significantly from the 2018 NICHD definition (z = 4.448, P < 0.001) and the 2019 Jensen definition (z = 4.428, P < 0.001). The highest AUC value corresponded to the model that the 2018 definition combined with birth weight, but there were no significant differences in AUC values among all the combined prediction models (P > 0.05).

## Discussion

In this study, two recently published BPD criteria, the 2018 NICHD and the 2019 Jensen definitions, were compared the predictive ability for death or severe respiratory morbidity in preterm infants with GA < 32 weeks at the corrected age of 18–24 months. Of the 451 infants included in the study, 141 (31.3%) had adverse outcomes. The logistic regression model and ROC analysis demonstrated that both diagnostic criteria exhibited good predictive ability for adverse outcomes, and the combination of gestational age or birth weight did not enhance their predictive value.

In our study, the criteria for the adverse outcomes were similar to those proposed by Jensen et al. [11] and Sun et al. [14]. However, due to the absence of tracheotomy cases in the infants we followed, this criterion was not considered in the determination of adverse outcomes. All 14 deaths in this study were associated with BPD. Moreover, 70.2% of the adverse outcome group have been rehospitalized for respiratory diseases ≥ 2 times, among 81.8% with BPD, indicating that BPD not only causes persistent lung injury and impedes alveolar and pulmonary vessel repair during the neonatal period, but also increases the susceptibility to respiratory infections in infancy and early childhood [15]. Nonetheless, we found that adverse outcomes were also observed in 10.6% of non-BPD infants, consistent with the findings of Jensen et al. (10%) [11]. This suggests that factors such as socioeconomic status, tobacco exposure, poor air quality, and maternal atopic disease and asthma may also contribute to an increased risk of late respiratory disease [16, 17]. Furthermore, our study further revealed that the risk of adverse outcomes significantly increased with increasing BPD severity according to the 2018 NICHD and 2019 Jensen definitions. This finding highlights the association between BPD severity and poor long-term respiratory prognosis [18].

In the univariate analysis, several factors were found to be statistically significant with adverse outcomes, including gestational age, birth weight, BPD, EUGR at 36 weeks’ PMA, and treatment with pulmonary surfactant. The incidence of BPD is known to increase as gestational age and birth weight decrease [19], which in turn can lead to long-term adverse respiratory events. Therefore, improving perinatal care to avoid the birth of extremely preterm and extremely low birth weight infants can be highly beneficial in reducing the incidence of adverse outcomes. Infants with BPD require increased energy to support their respiratory muscle workload, however, fluid restriction may result in inadequate energy intake leading to EUGR, which can impede lung and pulmonary vessel development and repair. The risk of EUGR is positively associated with the severity of BPD in preterm infants [20]. Our study revealed that EUGR at 36 weeks’ PMA was associated with poor long-term prognosis, emphasizing the importance of strengthening perinatal and enteral nutrition management after birth to prevent long-term adverse outcomes.

The 2001 NICHD definition has been widely utilized in clinical practice for the past two decades, but its identification of mild BPD offers limited predictive value for long-term prognosis [3]. In addition, a prior study indicated that the 2001 NICHD definition failed to consider advancements in respiratory support and expanded the diagnosis of BPD, resulting in an underestimation of mortality for severe BPD [21]. Consequently, the 2001 NICHD definition is no longer applicable for clinical practice, consistent with Poindexter et al.‘s conclusions [12]. In contrast, the two updated diagnostic criteria incorporate all noninvasive support modalities. The 2018 NICHD definition provides a comprehensive and detailed integration of the mode of respiratory support and FiO_2_, whereas the 2019 Jensen definition simplifies clinical practice and research by solely focusing on the respiratory support administered at 36 weeks’ PMA, regardless of current level of oxygen therapy.

Upon comparing the predictive models of the 2001 NICHD, 2018 NICHD, and 2019 Jensen definitions, we found that both novel diagnostic criteria were effective in predicting adverse outcomes and outperformed the 2001 NICHD definition. This finding supports that of Perez-Tarazona et al. [22], despite differences in the study population and adverse outcome indicators. They only explored the prognosis of infants with BPD, but our study included all preterm infants meeting the inclusion criteria. Although a higher grade of BPD is associated with an increased likelihood of adverse outcomes, relying solely on grade 3 BPD to predict prognosis is not sufficiently effective. However, the 2018 definition of grade 3 BPD outperformed the 2019 definition in predicting prognosis, possibly attributed to its inclusion of infants requiring oxygen flow rates ≥ 3 L/min and FiO_2_ ≥ 30% of noninvasive ventilation, in addition to those with invasive ventilation with FiO_2_ > 21% at 36 weeks’ PMA [10]. Previous studies have indicated that respiratory diseases are prevalent among preterm infants, even in the absence of BPD, primarily due to their gestational age and birth weight [23–25]. Our study found that the specificity of gestational age or birth weight alone in predicting adverse outcomes was lower compared to the diagnostic criteria of BPD. Moreover, combining these factors with BPD did not improve the predictive value of BPD diagnostic criteria. Therefore, BPD as a clinical diagnosis suggests that these preterm infants are at high risk for adverse pulmonary outcomes [26].

In this study, the AUC of the 2019 Jensen definition was 0.770, which was similar to that of the optimal definition derived from Jensen et al.‘s original data (AUC = 0.785). However, it should be noted that Jensen’s study primarily consisted of preterm infants with GA < 27 weeks, while the present study predominantly enrolled those born at 28–32 weeks’ gestation. So our study might further verify that the 2019 Jensen definition is also applicable in predicting the long-term outcomes of more mature preterm babies. The 2018 definition takes into account the oxygen concentration, which is subject to a certain degree of subjectivity and may impact the grading evaluation of BPD. In contrast, the 2019 definition only considers the mode of respiratory support, which is more objective and better suited for clinical application. Therefore, the 2019 definition provides a more accurate grading of BPD based on the mode of respiratory support at 36 weeks’ PMA, enabling early identification of infants with poor respiratory prognosis and facilitating timely interventions.

There were some limitations in our study. The primary limitation is that the population’s gestational age was 29.9 (28.4, 31.0) weeks, and the proportion of infants with GA < 27 weeks was relatively small (7.1%) compared to Jensen’s study. Therefore, caution should be exercised when generalizing these findings to populations with a wider range of gestational ages. Additionally, the follow-up period was limited to 18–24 months of corrected age, and further long-term studies are necessary to comprehensively evaluate the outcomes of preterm infants with BPD.

In conclusion, both novel definitions of BPD show comparable predictive values for death or severe respiratory morbidity through 18–24 months’ corrected age, with superior specificity; however, their combination with gestational age or birth weight does not enhance their predictive abilities. This study provides valuable guidance for selecting clinical diagnostic criteria and establishes a theoretical foundation for developing future diagnostic criteria. Further researches are required to propose a comprehensive definition based on pathophysiology for early identification of severe BPD.

### Electronic supplementary material

Below is the link to the electronic supplementary material.

Supplementary Material 1

## Data Availability

The datasets used and/or analysed during the current study are available from the corresponding author on reasonable request.

## Abbreviations

AUC: Area under the curve
BPD: Bronchopulmonary dysplasia
EUGR: Extrauterine growth restriction
FiO_2_: Fraction of inspired oxygen
GA: Gestational age
NICHD: National Institute of Child Health and Human Development
PMA: Postmenstrual age
ROC: Receiver operating characteristic
