# Supplementary material for: Comparison of two novel diagnostic criteria for bronchopulmonary dysplasia in predicting adverse outcomes of preterm infants: a retrospective cohort study
Source: BMC Pulm Med. 2023 Aug 23;23:308. doi: 10.1186/s12890-023-02590-6 (PMC10464144; doi:10.1186/s12890-023-02590-6)

Additional Files

Comparison of two novel diagnostic criteria for bronchopulmonary dysplasia in predicting adverse outcomes of preterm infants: a retrospective cohort study

Table S1. Predictive values of different diagnostic criteria and comparative analysis of AUC

Figure S1. ROC curves for evaluating adverse outcomes using different diagnostic criteria.

Table S1. Predictive values of different diagnostic criteria and comparative analysis of AUC

Variables	Sensitivity(%)	Specificity(%)	AUC	95%CI	z-*value*	*P-value*
2001 NICHD	77.3	65.2	0.730	0.689-0.778		
2018 NICHD	36.2	93.5	0.771	0.724-0.818	3.958[a]	<0.001
2019 Jensen	21.3	96.8	0.770	0.723-0.818	3.241[a]	0.001

a: Compared with 2001 NICHD definition.

Figure S1. ROC curves for evaluating adverse outcomes using different diagnostic criteria.

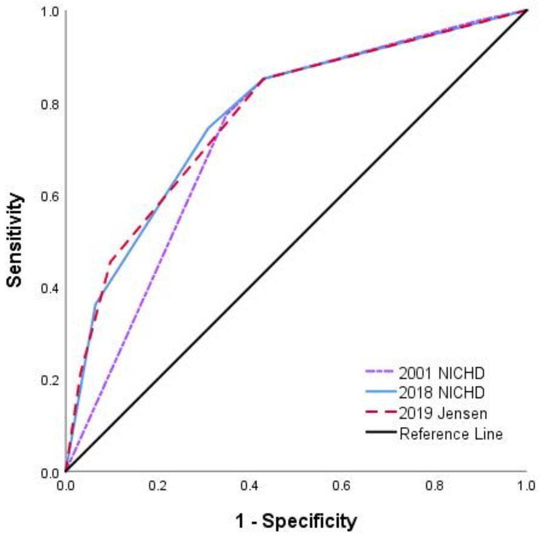

Supplement: Supplementary file 1 — Supplementary Material 1 [file 12890_2023_2590_MOESM1_ESM.pdf]
